# Morphological and molecular identification of fungi isolated from spoilt apples in Ota metropolis

**DOI:** 10.1186/s12866-025-04079-0

**Published:** 2025-06-07

**Authors:** Emmanuel O. Olumuyiwa, Mobolaji T. Ajetunmobi, Omolara F. Adeniji, Adewale K. Ogunyemi

**Affiliations:** 1 Department of Biological Sciences, Microbiology unit, Bells University of Technology, Ota Ogun-State, Nigeria; 2 Department of Biological Sciences (Microbiology Unit), Trinity University, Yaba, Lagos State Nigeria

**Keywords:** Identification, Spoilage, Apples, Characterization, Fungi, Primers

## Abstract

Spoilage of apples continues to be a significant issue in the fruit industry. This study aimed to isolate and identify fungal species on deteriorated apples collected from three different locations in Ota market, Ota, Ogun State, Nigeria. A total of eighteen (18) samples of red delicious and Granny Smith apples with obvious spoilage were collected, and their surfaces were sterilized using 85% ethanol. After that, the samples were cultivated on potato dextrose agar (PDA) supplemented with 30 mg/l of chloramphenicol, and incubated at 30 °C for five to seven days. From the subcultures of the primary plates, pure fungal cultures were obtained and were identified by morphological characterization and internal transcribed spacer (ITS1/ITS4) gene method. Ten fungi that cause spoilage in apples have been identified and grouped into six distinct classes. Among the 40 isolates, the most common one was *Trametes polyzona* strain MT9, accounting for 27.5% of the total isolates. The second most prevalent isolate was *Geotrichum candidum* strain MT10, with six isolates, representing 15% of the total. The least frequent was *Fusarium* sp. strain MT3, with only one isolate, amounting to 2.5%. It was in this connection, that a sequence analysis of the ITS regions of the nuclear-encoded rDNA was conducted, revealing significant alignments with *Aspergillus* sp., *Lasiodiplodia theobromae*, *Curvularia aeria*, and *Trametes polyzona*. This research investigation sought to elucidate the relationships between specified species, yielding a biocontrol strategy for mitigating fruit deterioration and conserving quality.

## Introduction

Fungi play an important contribution in nutrition, medicine, and biocontrol of plant pathogens. Certain yeasts, for example, act as antagonists in postharvest infections on fruits especially apples and citrus [11, 45]. However, fungi cause most plant diseases, making for perhaps 70% of all major crop diseases [45]. Besides the effects of high temperature and relative humidity, fungi produce pectic enzymes that break down apple pectin to release the nutrients of the cells to the fungi [11, 34].

According to Al-Hindi et al. [1], apples serve as a pivotal component of human nutrition, providing indispensable micronutrients, including vitamins and minerals, necessary for optimal health. However, they become insufficient because of agricultural losses in the field, during storage, transportation, or transshipment, and handling procedures from the grower to the wholesale dealer and retailer, and ultimately to consumers [17, 117]. Fruits are easily infected by microorganisms due to their succulent nature. Many kinds of microbes can grow and survive successfully on the good substrate that the high concentration of different carbohydrates, minerals, vitamins, and amino acids offers [10].

The most common causes of apple rot are from the fungi *Penicillium expansum*, and *Monilinia fructigena* [29, 43]. Other fungal genera isolated from apples include *Colletotrichum*, *Xylaria*, *Botryosphaeria* [15], and *Rhizopus oryzae* [56]. *Aspergillus* spp. has also been isolated and known to cause infections or allergies [73]. In some studies, *Cladosporium* spp. was found to be a frequent fungus found in stored apples, and *Penicillium*, *Acremonium*, *Aspergillus*, *Aureobasidium*, *Cryptococcus*, *Sporobolomyces* and *Alternaria* spp. [9, 86, 111]. Studies on fungal practices have traditionally involved conventional culture and microscopic identification [41]. Based on morphological traits (morphology, conidia size, and morphology of conidiophore) and mycelia (color, size, and shape), fungal species can be identified [1, 81]. Expert taxonomists are required for these techniques. Due to slight differences in the medium composition, mycelia features can be challenging to compare well [57]. It has been demonstrated that identifying fungi using molecular methods is an easy and effective process. DNA-based assays provide a reliable way to identify a wide variety of fungi. Numerous molecular approaches have been used to identify *Aspergillus* in clinical and environmental samples [26, 59, 64].

*Aspergillus* has been detected at the genus level using the 18S rRNA gene, mitochondrial DNA, the intergenic spacer region, and the internal transcribed spacer (ITS) regions as targets. Because there are roughly 100 copies of the ITS sections per genome, they provide unique benefits over other molecular targets. The ITS regions are situated between the 18S and 28S rRNA genes. ITS regions are used in phylogenetic analyses of a wide variety of organisms due to their sequence variation [6]. Other research [39, 40] examined whether there was enough variety in the clinically significant *Aspergillus* species'ITS 1 and 2 nucleotide sequences to allow for species-level identification.

Nonetheless, Ota City has paid little to no care to preventing fungi from spoiling apple fruits. Because of this, the frequency of the fungal attacks on the fruits has necessitated a specific analysis of current research to isolate and identify fungi that cause fruit spoiling on apples and to identify the fungal isolates molecularly.

## Materials and methods

### Samples from Ota market

#### Red delicious apples

This apple variety has thick, bright red skin. The peel is edible, crisp, and slightly bitter. Farmers favor this popular cultivar over local varieties. Compared to rounder types, the Red Delicious apple is thinner in shape.

#### Granny Smith apples

Granny Smith apples offer a perfect balance of sweetness and tartness. Their vibrant green color and hardy nature help them stay fresh during shipping. When stored in cold conditions, these apples can last up to six months.

#### Preliminary survey/experimental design and sample collection

The preliminary study was carried out to establish and identify where to acquire the apple samples for this research. However, an experimental design was adopted in the following pattern. The six samples exposed under the sun in the market included three Granny Smith apples and three Red Delicious apples. Six (6) apple samples (three (3) Granny Smith apples and three (3) Red delicious apples) × 1 retailer × 3 markets (Toll Gate, Oju-Ore, and Sango Otta), with their decimal degrees Co-ordinates: N6.688381, E3.261913; N6.688497, E3.227337; N6.607506, E3.243139 respectively. A total of nine (9) apples of the Granny Smith variety and nine (9) apples of the red delicious variety were collected and used for this study and were divided into three (3) groups or locations based on purchase. The apples had visible lesions or spoilage and were placed in separate sterile plastic bags before being transported to Bells University Microbiology Laboratory within an hour of collection. At the laboratory, the fungi in each sample were identified.

#### Fungal isolation and purification

There are many different species of apple fruits. The diseased samples were sliced from the expanding edges of the lesion using a sterile knife. The cut portion of the lesion was disinfected with ethanol (85%) for 2 min. These were then rinsed in three different changes of distilled water. Each portion was then homogenized using a sterile glass rod and a test tube containing 10 ml of the homogenate (1 g + 9 ml) (10^1^) was made and serially diluted down to 10^–4^. Plates of already prepared sterile Potato Dextrose Agar (PDA) containing Chloramphenicol (30 mg/l) to prevent the growth of bacteria were inoculated with 0.1 ml aliquots of the serially diluted samples and incubated at ambient room temperature (25–30 °C) for seven days. After seven days, the growth of fungal colonies on PDA was counted in triplicate and recorded in a colony-forming unit per gram (cfu/g). The fungal colonies were observed, and the pure cultures were maintained [33, 46, 47]. Isolated species were sent for molecular confirmation.

#### Macroscopic and microscopic examination of isolated fungi

The fungal morphology was studied macroscopically by observing the colony features (colour, shape, size, and hyphae), and microscopically by a LED Binocular compound laboratory microscope (OMAX 40x-200x, China) using a lactophenol cotton blue-stained slide mounted with a small portion of the mycelium [33].

### Molecular identification of fungal species

#### DNA extraction and PCR amplification

The DNA Extraction of genomic DNA from the fungi was conducted from a one-week-old PDA culture using a Zymo Fungal DNA extraction kit. The purity and concentration of the extracted DNA were evaluated using a NANODROP (ND 1000) Spectrophotometer (Thermo Scientific, USA). All the samples showed a DNA yield between 5 ng—25 ng, and the extracted DNA was optimally pure showing A260/A280 between 1.60–1.80. Primers ITS-1 (5'-TCCGTAGGTGAACCTGCGG) and ITS-4 (5'-TCCTCCGCTTATTGATATGC) were used to amplify ribosomal internal transcribed spacer (ITS). PCR products were purified using the QIA quick PCR purification kit (QIAGEN, GmbH, Germany) [4].

PCR was performed in 25 µl of a reaction mixture, and the reaction concentration was brought down from 5 × concentration to 1X concentration containing 1X Blend Master mix buffer Buffer (Solis Biodyne), 1.5 mM MgCl2, 200 µM of each deoxynucleoside triphosphates (dNTP)(Solis Biodyne), 25pMol of each primer (BIOMERS, Germany), 2 unit of Hot FIREPol DNA polymerase (Solis Biodyne); however, additional Taq DNA polymerase was incorporated into the reaction mixture to make a final concentration of 2.5 units of Taq DNA polymerase, Proofreading Enzyme, 2 µl of the extracted DNA, and sterile distilled water was used to make up the reaction mixture.

Thermal cycling was conducted in an Eppendorf Vapo protect thermal cycler (Nexus Series) for an initial denaturation of 95 °C for 15 min followed by 35 amplification cycles of 30 s at 95 °C; 1 min at 58 °C and 1 min 30 Seconds at 72 °C. This was followed by a final extension step of 10 min at 72 °C. The amplification product was separated on a 1.5% agarose gel and electrophoresis was carried out at 80 V for 1 h 30 min. After electrophoresis, DNA bands were visualized by ethidium bromide staining. 100 bp DNA ladder was used as DNA molecular weight standard.

#### Sequencing and Bayesian phylogenetic analysis

The PCR products were submitted to Epoch Life Science (USA) for Sanger sequencing. The obtained ITS gene sequences were initially analyzed using the BLAST program (National Center for Biotechnology Information [NCBI], https://www.ncbi.nlm.nih.gov/BLAST/) and subsequently deposited in GenBank to obtain accession numbers. Bayesian phylogenetic analyses were conducted to compare the fungal isolates with selected reference strains from global databases. The optimal nucleotide substitution models for each gene dataset were selected using jModelTest [21], with model selection based on the corrected Akaike Information Criterion (AICc). The Kimura 2-parameter model with a discrete Gamma distribution (K2 + G) was applied to the sequence data. Bayesian inference was performed using MrBayes v3.2.7 [87], with Monte Carlo Markov Chain (MCMC) parameters set to ten million generations and sampling every 1,000 generations. Two independent runs were conducted per analysis, with a minimum probability threshold of 0.05. Prior to convergence, a 25% relative burn-in was applied to remove early unstable trees for diagnostic assessment. Chains were heated to a temperature of 0.10, and the resulting phylogenetic trees were visualized using FigTree v1.4.4 [83].

## Results

### Description of fungi symptoms on apple fruits

**Fig. 1:**
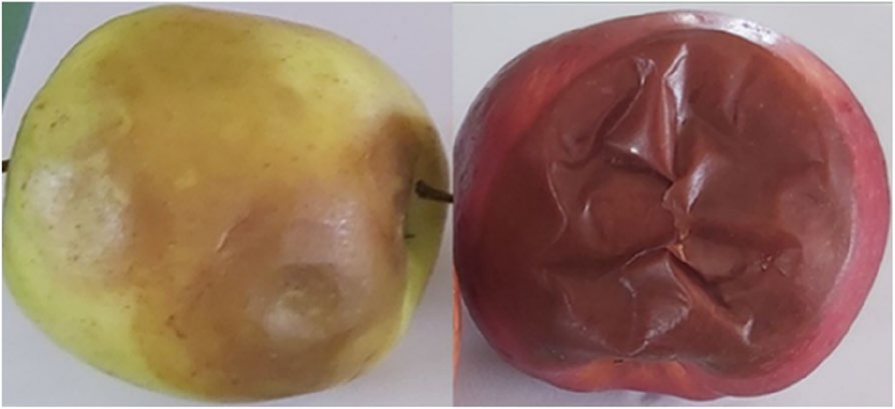
Description of fungi disease symptoms on apple fruits

Symptoms of microbial contamination occurred in the form of necrosis of soft rots reddish, blackish, whitish, greenish, or grayish colour with or without openings and also the presence of round spots (Fig. 1).

### Isolation of fungal strains

From the infected fruits (Granny Smith and red delicious apples), 35 strains of fungi were isolated. The analysis of the morphology characterization, after isolation and purification of the isolates, gave ten (10) morphological groups different from each other based on their appearances, densities, colours, sizes, and mycelia (Table 1). The ten (10) fruit spoilage fungi were identified, designated, and assigned accession numbers as *Aspergillus tubingensis* strain MT1 (OR501379), *Aspergillus brunneoviolaceus* strain MT2 (OR501380), *Fusarium* sp. strain MT3 (OR501381), *Aspergillus* sp. strain MT4 (OR501382), *Blakeslea trispora* strain MT5 (OR501383), *Penicillium* sp. strain MT6 (OR501384), *Lasiodiplodia theobromae* strain MT7 (OR501385), *Curvularia aeria* strain MT8 (OR501386), *Trametes polyzona* strain MT9 (OR501387), and *Geotrichum candidum* strain MT10 (OR501388) respectively*.*

**Table 1:** Macro and Micro morphologies of different fungal strains from spoilt apples

Strains code	Fungal specie	Macroscopic view on PDA	Microscopic view
MT1	*Aspergillus tubingensis*		
MT2	*Aspergillus brunneoviolaceus*		
MT3	*Fusarium* sp.		
MT4	*Aspergillus* sp.		
MT5	*Blakeslea trispora*		
MT6	*Penicillium* sp.		
MT7	*Lasiodiplodia theobromae*		
MT8	*Curvularia aeria*		
MT9	*Trametes polyzona*		
MT10	*Geotrichum candidum*		

*PDA* Potato Dextrose Agar, *LPB* Lacto Phenol Blue

### Macroscopic and microscopic identification of fungal strains

In this study, the isolated fungal strains were examined based on cultural, microscopic, and morphological characteristics, and their presumptive identification was determined (Table 2). The total samples obtained and the mean viable fungal count (cfu/g) obtained per market location are presented in Table 3. Furthermore, the analysis of variance (ANOVA) procedure to determine variation between markets and variation between apples showed that the probability value for markets variation is 0.114 while the probability value for apples variation is 0.826. Consequently, there is no variation in the total fungal count between the three markets at 5% level of significance and also there is no variation in the total fungal counts between apples at 5% level. The results suggest that fungal count between markets and apples are the same. A total of eighteen (18) samples were collected from three (3) different sampling market points or locations. The result showed that the Oju-Ore and Sango Otta markets of Red Delicious apples and Granny Smith apples had 3 (100%) of the samples infected. In comparison, Tollgate market of red delicious apples had 2 (66.7%) infected. The mean fungal count ranged between 1.0 × 10^3^ and 4.5 × 10^3^ cfu/g respectively. In the percentage frequency of occurrence, *Trametes polyzona* strain MT9 had the highest occurrence of 27.5%, followed by *Geotrichum candidum* strain MT10 (15%), *Aspergillus* sp. strain MT4 and *Curvularia aeria* strain MT8 (10%), *Aspergillus brunneoviolaceus* strain MT2, *Blakeslea trispora* strain MT5, *Penicillium* sp. strain MT6, and *Lasiodiplodia theobromae* strain MT7 (7.5%) each, with *Fusarium* sp. strain MT3 having the least of occurrence of 2.5% (Table 4). *Trametes polyzona* was the most frequent isolate, obtained in 3 of a total of 15 isolates (27.5%), followed by *Geotrichum candidum* and *Curvularia aeria* with three isolates (15%) and two isolates (10%) respectively, and the least common was *Fusarium* sp. with one isolate (2.5%). *Aspergillus* species consisted of a total of 3 (22.5%) isolates (Table 4).

**Table 2:** Characterization of the fungal isolates isolated from fruits on potato dextrose agar (PDA)

Strains code	Macroscopic Culture characteristics	Microscopic Characteristics	Name of fungal isolates
MT1	Colonies are granular, velvety, or wooly and yellow–brown	Philades are circumferential and are biseratial	*Aspergillus tubingensis*
MT2	Large black head (thick black) colonies	Septate hyphae with non-septate conidiophore showing mop-like conidial head bearing spherical	*Aspergillus brunneoviolaceus*
MT3	A cotton colony mycelium sparse becoming gray with maturity with a yellow-whitish center	Revealed sickle-shaped macroconidia	*Fusarium* sp.
MT4	Colonies are grayish-white wooly and fluffy growth	Chains of macroconidia	*Aspergillus* sp.
MT5	Yellow-orange colonies	Short apical sporangiophore with bearing few spored sporangia	*Blakeslea trispora*
MT6	Greenish colonies with radiated white ring	Repeatedly branched conidiospores of long chains on conidia	*Penicillium* sp.
MT7	Dark gray aerial mycelia	Septate fungal hyphae with acropetal long chains of conidia	*Lasiodiplodia theobromae*
MT8	Smoke grey to olivaceous black, with moderate aerial mycelium giving the colony a cottony appearance, margin fimbriate to lobate	Brown septate hyphae and conidia (spores) with a blue/purple colour	*Curvularia aeria*
MT9	Appearance of milky foam/curd	Profused blue-colored hyphae, spores, and pores of the fungus	*Trametes polyzona*
MT10	Creamy and cottony white	Blue-stained hyphae, spores, and arthroconidia of the fungus	*Geotrichum candidum*

**Table 3:** Samples of apparently diseased apples and the mean viable fungal count from Ota market, Ogun-State

Kinds of apples	Total samples obtained per market location	Total samples infected	% samples infected	TFC (cfu/g)
Red delicious apples	3 (a)	2	66.7	4.0 × 10^3^
	3 (b)	3	100	1.0 × 10^3^
	3 (c)	3	100	2.5 × 10^3^
Granny Smith apples	3 (a)	3	100	4.5 × 10^3^
	3 (b)	3	100	1.5 × 10^3^
	3 (c)	3	100	1.0 × 10^3^

^a^Tollgate market

^b^Oju Ore market

^c^Sango Otta market

**Table 4:** Total count, frequency of occurrence of various fungal strains, and percentage frequency from 18 samples on Potato Dextrose Agar (PDA) containing chloramphenicol (30 mg/l)

Strains	Genera specie	Total count	Frequency	% occurrence
MT1	*Aspergillus tubingensis*	2	1	5
MT2	*Aspergillus brunneoviolaceus*	3	1	7.5
MT3	*Fusarium* sp.	1	1	2.5
MT4	*Aspergillus* sp.	4	1	10
MT5	*Blakeslea trispora*	3	1	7.5
MT6	*Penicillium* sp.	3	1	7.5
MT7	*Lasiodiplodia theobromae*	3	1	7.5
MT8	*Curvularia aeria*	4	2	10
MT9	*Trametes polyzona*	11	3	27.5
MT10	*Geotrichum candidum*	6	3	15
**Total count**		40	15	100

### Molecular identification of the fungal strains

**Fig. 2:**
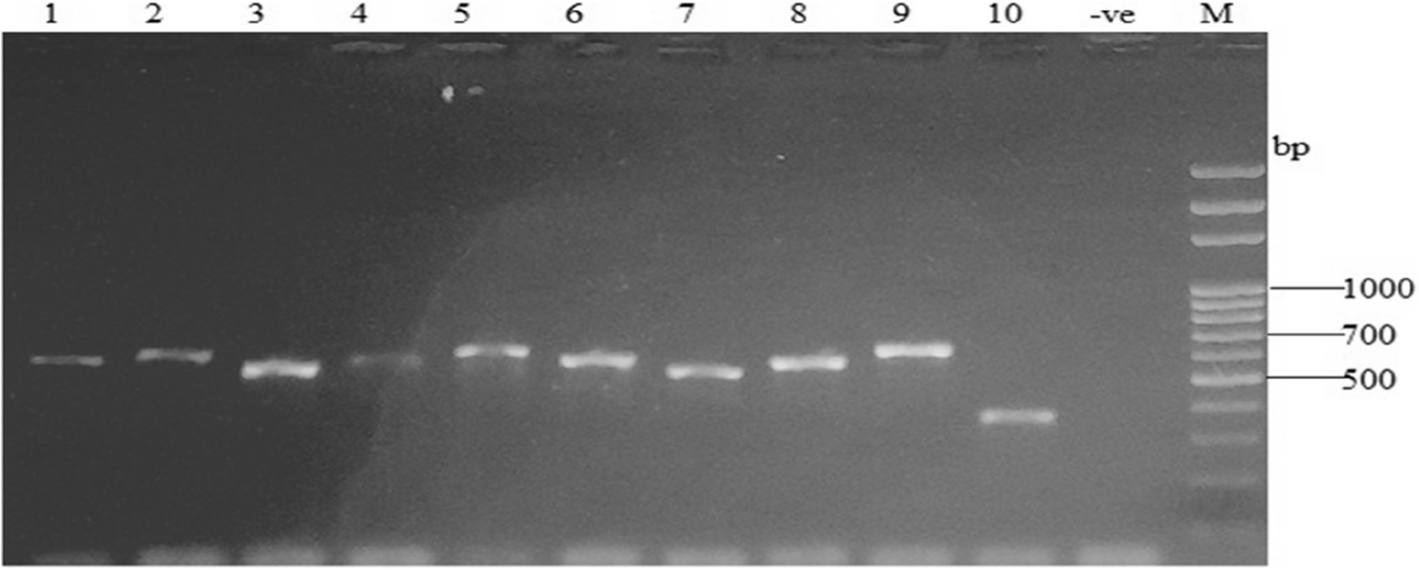
Gel electrophoresis of PCR of fungi DNA verification. Lane1, strain MT1; Lane 2, strain MT2; Lane 3, strain MT3; Lane 4, strain MT4; Lane 5, strain MT5; Lane 6, strain MT6; Lane 7, strain MT7; Lane 8, strain MT8; Lane 9, strain MT9; Lane 10, strain MT10; -ve, Negative control; bp, base pair and M, molecular marker

Strains were identified using the molecular method of 18S rRNA gene sequence analysis. The quality of the DNA was confirmed by PCR amplification of the fungal conserved 28S rDNA region using the primer pair ITS-1 (5'-TCCGTAGGTGAACCTGCGG) and ITS-4 (5'-TCCTCCGCTTATTGATATGC) with control DNA from pure strains. The PCR products of amplified ITS genes provided unique, unambiguous, and intense bands between 400 and 600 bp, which corresponded to the expected size between 300 and 700 bp for fungi (Fig. 2). The negative control was performed with the reaction mixture without the addition of DNA extract. The absence of a band for the negative control showed that there was no contamination of the PCR reaction mixture. After electrophoresis, the bands obtained from 1.5% agarose gel were finally sequenced and identified using the online blast search at http://blast.ncbi.nlm.nih.gov/Blast.cgif for strain identification. Identification of strains revealed ten (10) distinct species such as *Aspergillus tubingensis*, *Aspergillus brunneoviolaceus*, *Fusarium* sp., *Aspergillus* sp., *Blakeslea trispora*, *Penicillium* sp., *Lasiodiplodia theobromae*, *Curvularia aeria*, *Trametes polyzona*, and *Geotrichum candidum* respectively.

### rDNA sequences’ analysis

**Fig. 3:**
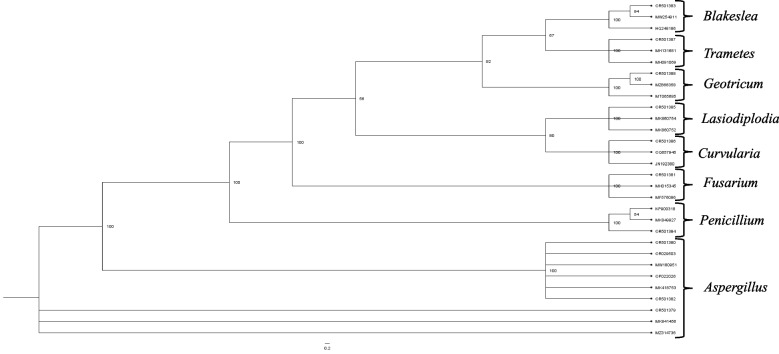
Bayesian phylogenetic analyses of fungal isolates with selected reference worldwide strains

Sequence analysis of the internal transcribed spacer (ITS) regions of the nuclear-encoded rDNA showed significant alignments of 93–100% with the isolated fungal species (Table 5). Figure 3 shows the Bayesian phylogenetic analyses which were conducted to compare the fungal isolates with selected reference strains from global databases. The numbers above tree branches represent Bayesian inference posterior probability.

**Table 5:** Identification of fungal isolates of ITS region of rRNA gene sequence

Strains	Species identified	Length (bp)	Identity (%)
MT1	*Aspergillus tubingensis*	563	99.64
MT2	*Aspergillus brunneoviolaceus*	533	99.81
MT3	*Fusarium* sp.	532	99.39
MT4	*Aspergillus* sp.	539	100
MT5	*Blakeslea trispora*	567	99.63
MT6	*Penicillium* sp.	552	99.05
MT7	*Lasiodiplodia theobromae*	1015	100
MT8	*Curvularia aeria*	1115	100
MT9	*Trametes polyzona*	650	100
MT10	*Geotrichum candidum*	325	92.71

## Discussion

Microbial contamination in apples manifests as necrosis, soft rot, and chromatic alterations (erythema, melanization, leukosis, or graying), often without visible entry points. These symptoms align with fungal diseases documented in tropical fruits [16] and are consistent with recent reports of postharvest apple spoilage caused by *Penicillium*, *Alternaria*, and *Lasiodiplodia* spp. [2]. While earlier studies focused on *Aspergillus niger* in citrus [8] and *Colletotrichum gloeosporioides* in mangoes [76], contemporary research highlights emerging pathogens like *Curvularia aeria* and *Lasiodiplodia theobromae* in apples, driven by climate-induced shifts in fungal ecology [68, 90].

In this study, ten fungal species were isolated from spoiled apples at the Ota market in Nigeria using both morphological and molecular methods (ITS-rDNA barcoding). Key isolates include Eurotiomycetes (*Aspergillus tubingensis*, *A. brunneoviolaceus*, and *Penicillium* spp.), Dothideomycetes (*Lasiodiplodia theobromae* and *Curvularia aeria*), Zygomycetes (*Blakeslea trispora*), and Saccharomycetes (*Geotrichum candidum*). Notably, *Trametes polyzona* (Agaricomycetes) was the most prevalent (27.5%), followed by *G. candidum* (15%) and *Fusarium* spp. (2.5%). Recent studies have confirmed *Lasiodiplodia* and *Curvularia* as aggressive postharvest pathogens in apples, with *L. theobromae* causing stem-end rot under humid storage conditions [102]. Many of these isolated fungi are classified within the Ascomycota, with one exception, *Blakeslea trispora*, belonging to the Zygomycota. Recent studies highlight that the exceptional spore abundance and efficient dispersal mechanisms of Ascomycetes have played a crucial role in their global distribution and evolutionary diversification [50, 82]. Advances in genomic and aerobiological research further suggest that adaptive spore traits, such as enhanced resilience and aerodynamic properties, contribute to their ecological success [85, 88].

The fungal isolates in this study were initially identified to the genus level through morphological characterization, including colony color assessment (both obverse and reverse sides) and microscopic examination of spore-producing structures. While traditional morphological methods remain useful for taxonomic classification at the family or genus level [110], their limitations in resolving species-level diversity are well-documented [66]. Recent studies emphasize the need for integrated approaches, combining morphological data with molecular techniques such as ITS sequencing or whole-genome analysis for accurate species delineation [19, 99, 107]. Advances in high-throughput sequencing and phylogenetic analyses have further highlighted the discrepancies between morphological and genetic classifications, underscoring the importance of polyphasic taxonomy in modern fungal systematics [44, 116].

Ten (10) fungal species were identified through DNA barcoding, with sequence identity ranging from 93 to 100%. The Internal Transcribed Spacer (ITS) region of ribosomal DNA (rDNA) remains the gold standard for fungal species identification, particularly in environmental samples [75, 91]. Recent studies have reinforced the utility of ITS sequencing in characterizing soil fungal communities with high resolution, outperforming traditional morphological methods [65, 99]. The ITS region is favoured for phylogenetic analyses due to its universal distribution, functional conservation, and sufficient variability to discriminate closely related species [18, 61]. Advances in high-throughput sequencing and bioinformatics have further enhanced ITS-based fungal diversity assessments, enabling more accurate taxonomic assignments and ecological insights [67].

Recent studies continue to identify *Penicillium* and *Aspergillus* species as predominant fungal pathogens contributing to fruit spoilage [25, 31, 98]. However, our findings contrast with earlier reports by Alwakeel [5], which highlighted different species, including *P. chrysogenum*, *P. adametzii*, and *A. oryzae*. Notably, *P. chrysogenum* (formerly *P. notatum*) has been detected in salted foods and water-damaged indoor environments [89, 106]. While *Penicillium* species are generally considered low-risk human pathogens, they remain invaluable for their role in β-lactam antibiotic production, particularly penicillin [14, 71]. Advances in genomic studies have revealed that enhanced penicillin biosynthesis in *P. chrysogenum* is linked to upregulated expression of genes involved in valine, cysteine, and α-aminoadipic acid metabolism, as well as peroxisomal protein synthesis [35], van den [104].

Despite its industrial benefits, *P. chrysogenum* has been increasingly recognized as an opportunistic pathogen in immunocompromised individuals. Recent case reports have associated it with invasive pulmonary infections, particularly in transplant recipients and HIV patients [49, 53]. Additionally, emerging evidence suggests its potential role in systemic infections, including disseminated mycosis in immunosuppressed hosts [7, 42].

*Curvularia aeria* represents a genetically and ecologically diverse species complex, encompassing both pathogenic and saprobic lineages with a broad host range across multiple plant taxa [68]. Recent studies highlight its pathogenic adaptability, not only causing foliar diseases in key bioenergy crops like switchgrass (*Panicum virgatum*) but also emerging as an opportunistic pathogen in human infections [20, 55]. Beyond its clinical and agricultural impacts, *Curvularia* species exhibit significant biotechnological potential, including applications in bioenergy production (e.g., biogas and biodiesel), heavy metal biosorption, and even uranium bioremediation [70, 92].

*Blakeslea trispora* is a biotechnologically significant fungus widely utilized for the industrial production of high-value carotenoids, particularly β-carotene and lycopene, which are employed as natural food colorants, nutraceuticals, and antioxidants [80]. Recent studies have optimized its biosynthetic pathways through metabolic engineering and fermentation strategies [63, 109]. Advances in strain improvement, substrate utilization, and light-regulation mechanisms have further enhanced its productivity, positioning *B. trispora* as a sustainable alternative to synthetic carotenoid production [36, 51].

*Aspergillus tubingensis*, distinguished by its limited mycotoxin production and robust enzymatic profile, has emerged as a promising candidate for biotechnological and industrial applications [3, 77]. Recent studies highlight its extensive enzymatic repertoire, including amylase, lipase, glucose oxidase, phytase, xylanase, acid phosphatase, and xylosidase, which enable diverse bioconversion processes [28]. Notably, its amylolytic activity enhances the hydrolysis of complex carbohydrates in agro-industrial byproducts such as distilled wastewater and molasses residues, improving bioethanol fermentation yields [93, 112]. Beyond biofuel production, *A. tubingensis* exhibits metabolic versatility in synthesizing high-value organic acids, including citric acid, ascorbic acid, and wood preservatives, at industrially viable scales [77, 79]. Recent advances also underscore its role in plastic waste management, as its enzymatic machinery facilitates polyurethane biodegradation through hydrolytic and oxidative pathways [52, 100]. In food biotechnology, the glucose oxidase (GOD) activity of *A. tubingensis* enhances dough rheology, improving bread texture, volume, and loaf structure [62]. Additionally, it contributes to traditional fermentation processes, such as in Chinese pu’er tea production, where it aids in the bioconversion of polyphenols into bioactive theobromins [108, 114].

*Lasiodiplodia theobromae* is a globally distributed phytopathogenic fungus with a broad host range, known for causing necrotic diseases such as stem-end rot in citrus, bot canker in *Vitis vinifera* [101], and wood lesions in *Biancaea sappan* (Sappanwood). Recent studies highlight its increasing prevalence as an emerging pathogen in tropical and subtropical regions, linked to climate change and agricultural intensification [69]. Beyond its phytopathogenicity, *L. theobromae* acts as an opportunistic human pathogen, implicated in rare cases of fungal keratitis, onychomycosis, and subcutaneous phaeohyphomycosis [37, 97].

In contrast, *Trametes polyzona*, a tropical white-rot fungus, has gained attention for its enzymatic potential in biotechnology. It secretes ligninolytic enzymes such as laccase, manganese peroxidase, and lignin peroxidase, which are valuable for bioremediation, biofuel production, and waste valorization [38, 94]. Recent advances in fungal biotechnology have optimized its enzyme production through metabolic engineering, enhancing its industrial applicability [115].

*Geotrichum candidum*, a saprotrophic fungus, plays a key role in shaping the texture and flavor of surface-ripened cheeses such as Saint-Marcellin, where it promotes the formation of a uniform, white, velvety rind [13, 24]. Recent studies highlight its diverse enzymatic arsenal—including lipases, proteases, and aminopeptidases—which critically influences cheese flavor development by liberating free fatty acids, generating small peptides, and degrading bitter compounds, particularly in Camembert and other mold-ripened varieties [60, 96]. Notably, *G. candidum*'s aminopeptidase activity has been linked to the production of key volatile compounds, such as branched-chain aldehydes and sulfur-containing molecules, which contribute to the characteristic nutty, mushroom-like aroma of traditional Norman Camembert [23, 58]. Advances in metatranscriptomics have further elucidated strain-specific metabolic contributions, revealing how *G. candidum* interacts with other ripening microbes to modulate flavor complexity [72].

Immunocompromised individuals face a heightened risk of severe fungal infections, whereas most fungal species play essential roles in food fermentation, antibiotic production, and other biotechnological applications [12, 54]. Although fungal infections in healthy individuals remain uncommon, emerging evidence suggests that climate change and antifungal resistance may be increasing sporadic cases [30, 113]. Agricultural workers, particularly those handling crops or soil, are occupationally exposed to fungal pathogens and mycotoxins, necessitating improved workplace safety measures [78, 84]. Assessing the mycotoxin-producing potential of environmental fungi is crucial for risk stratification and the development of targeted public health interventions [27, 105].

Recent studies, including those by Mukhtar et al. [74], alongside earlier work by Uzuegbu and Emifoniye [103], highlight that the observed heterogeneity in fungal isolates is shaped by a complex interplay of factors such as storage conditions, product diversity, and regional variations in microflora linked to different fruit cultivation areas. Emerging research [25, 95] reinforces that farm-level contamination during harvesting remains the primary source of fungal spores, with secondary transmission occurring in storage facilities through cross-contamination from already infected fruits. This aligns with Jay’s [48] findings that most spoilage microorganisms responsible for post-harvest losses originate during harvesting operations. Further investigations [32, 93] emphasize that inadequate post-harvest management accelerates fungal proliferation, leading to significant economic losses and heightened health risks for consumers due to mycotoxin exposure. Advanced genomic studies have also identified region-specific fungal strains, underscoring the role of geographical factors in contamination patterns [22]. Without targeted interventions, these fungal pathogens continue to threaten food security and public health, necessitating improved sanitation practices and storage technologies to mitigate risks.

## Conclusion

The isolation and identification of filamentous fungi from three locations in Ota market revealed the prevalence of economically significant fungal species, highlighting both agricultural and public health concerns. Utilizing advanced molecular techniques, this groundbreaking study provided precise differentiation between closely related fungal species, surpassing the limitations of traditional morphological identification. The findings demonstrated that *Trametes polyzona*, *Geotrichum candidum*, and *Fusarium* sp. were the most predominant contaminants in spoiled apple fruits, with some samples exhibiting co-contamination by multiple fungi. Notably, *T. polyzona* and *G. candidum* are known mycotoxin producers, posing severe health risks to consumers upon ingestion. These findings present significant challenges for farmers and traders in ensuring safe, marketable produce. To mitigate fungal contamination, integrated management strategies—including proper harvesting techniques to prevent fruit damage, optimized storage conditions, and the application of plant-derived antifungal agents—are essential. This study also emphasizes the need for expanded research into filamentous fungi, particularly in taxonomy and pathogenicity, to enhance detection and control measures. Future investigations should explore the genomic diversity of these fungi and their adaptive mechanisms in postharvest environments, contributing to more effective biocontrol solutions and food security policies.

## Data Availability

The Sequence data that support the findings of this study have firstly been blasted using the BLAST program (http://www.ncbi. nlm. nih. gov/BLAST/) and deposited in the GenBank for accession numbers (OR501379, OR501380, OR501381, OR501382, OR501383, OR501384, OR501385, OR501386, OR501387, OR501388).
